# Acalculous Cholecystitis in COVID-19 Patients: A Narrative Review

**DOI:** 10.3390/v16030455

**Published:** 2024-03-15

**Authors:** Evanthia Thomaidou, Eleni Karlafti, Matthaios Didagelos, Kalliopi Megari, Eleni Argiriadou, Karolina Akinosoglou, Daniel Paramythiotis, Christos Savopoulos

**Affiliations:** 1 Department of Anesthesiology and Intensive Care Unit, AHEPA University General Hospital, Aristotle University of Thessaloniki, 54636 Thessaloniki, Greece; evathom1975@yahoo.gr (E.T.); manthosdid@yahoo.gr (M.D.); argiriadouhelena@hotmail.gr (E.A.); 2 Emergency Department, AHEPA University General Hospital, Aristotle University of Thessaloniki, 54636 Thessaloniki, Greece; linakarlafti@hotmail.com; 3 First Propaedeutic Internal Medicine Department, AHEPA University General Hospital, Aristotle University of Thessaloniki, 54636 Thessaloniki, Greece; 4 1st Cardiology Department, AHEPA University General Hospital, Aristotle University of Thessaloniki, 54636 Thessaloniki, Greece; 5 CITY College, University of York Europe Campus, 54626 Thessaloniki, Greece; kmegari@psy.auth.gr; 6 Department of Medicine, University General Hospital of Patras, 26504 Rio, Greece; karolina_akinosoglou@upatras.gr; 7 First Propaedeutic Department of Surgery, AHEPA University General Hospital, Aristotle University of Thessaloniki, 54636 Thessaloniki, Greece; danosprx1@hotmail.com

**Keywords:** acalculous cholecystitis, gallbladder, COVID, SARS-CoV-2

## Abstract

Acute acalculous cholecystitis (AAC) represents cholecystitis without gallstones, occurring in approximately 5–10% of all cases of acute cholecystitis in adults. Several risk factors have been recognized, while infectious diseases can be a cause of cholecystitis in otherwise healthy people. Coronavirus disease 2019 (COVID-19) is caused by the severe acute respiratory syndrome coronavirus 2 (SARS-CoV-2) and has spread worldwide, leading to an unprecedented pandemic. The virus enters cells through the binding of the spike protein to angiotensin-converting enzyme 2 (ACE2) receptors expressed in many human tissues, including the epithelial cells of the gastrointestinal (GI) tract, and this explains the symptoms emanating from the digestive system. Acute cholecystitis has been reported in patients with COVID-19. The purpose of this review is to provide a detailed analysis of the current literature on the pathogenesis, diagnosis, management, and outcomes of AAC in patients with COVID-19.

## 1. Introduction

Acute acalculous cholecystitis (AAC) was first described by Duncan in 1884 and represents cholecystitis without gallstones, being present in approximately 5–10% of all cases of acute cholecystitis in adults [1,2,3,4]. AAC occurs more frequently in critically ill patients, and risk factors include trauma, surgery, shock, burns, sepsis, total parenteral nutrition (TPN), and mechanical ventilation [3]. Unfortunately, it is associated with a high mortality rate of approximately 30%, not only because the disease itself is severe but also because it can be complicated by necrosis, perforation, and empyema with a higher incidence than calculous cholecystitis [3]. AAC can also appear in healthy people, mainly due to infectious causes [2,4].

Coronavirus disease 2019 (COVID-19) is caused by the severe acute respiratory syndrome coronavirus 2 (SARS-CoV-2) and was first reported in December 2019 in Wuhan, China. It spread worldwide, leading to a devastating pandemic with more than 300 million cases and more than 5 million deaths reported worldwide in 3 years [5,6]. The virus invades cells through the binding of the SARS-CoV-2 spike protein to angiotensin-converting enzyme 2 (ACE2) receptors expressed on the host cell surface. ACE2 receptors are expressed in many human tissues, with the highest expression levels being found in the alveolar cells of the lungs and the epithelial cells of the gastrointestinal (GI) tract, explaining the respiratory and gastrointestinal symptoms of the disease [7].

The purpose of this review is to provide a detailed analysis of the current literature on the pathogenesis, diagnosis, management, and outcomes of AAC in patients with COVID-19. An international literature search was carried out via MEDLINE/PubMed, Scopus, the Cohrane Library, and Google Scholar using the keywords “acute acalculous cholecystitis” and “COVID-19”. After title and abstract screening, all manuscripts describing at least one case of AAC in COVID-19 patients were further analyzed, and after duplicate removal, all cases were included in this review. 

## 2. Gastrointestinal Manifestations and Complications of COVID-19 Infection

Angiotensin-converting enzyme 2 (ACE2) receptors are largely expressed in the gastrointestinal (GI) tract, making it prone to direct damage from the cellular invasion of severe acute respiratory syndrome coronavirus 2 (SARS-CoV-2). Many GI symptoms have been described in patients with COVID-19 disease, with their incidence ranging from 2% to 79.1%. The most common symptoms include anorexia (26.8%), diarrhea (7.4–12.5%), nausea/vomiting (4.6–10.2%), and abdominal pain/discomfort (9.2%) [8,9,10,11,12,13]. More severe complications like ileus and life-threatening mesenteric ischemia arise in 74% to 86% of critically ill patients during lengthy hospitalization [14,15,16]. Certain GI illnesses (Inflammatory Bowel Disease, Chronic Liver Disease, Gastroesophageal Reflux Disease, Peptic Ulcer Disease, and GI malignancies) predispose for severe COVID-19 infection, while proton pump inhibitor (PPI) and glucocorticoid use have been associated with severe clinical outcomes in COVID-19 infection [8,17,18].

## 3. Biliary Manifestations of COVID-19

Acute cholecystitis has been reported in patients with COVID-19, primarily in those who are critically ill. Most cases are AAC, which occurs due to a reduction in the mobility of the gallbladder and occurs in patients with severe illness, infection, and mechanical ventilation [8]. Because of the widespread expression of ACE2 in the liver, gallbladder, and bile ducts, the virus may play a major role in the development of cholecystitis through direct invasion and replication in the gallbladder and bile ducts. The detection of viral RNA in the bile wall and gallbladder further supports this hypothesis. Other possible mechanisms include inflammation due to cytokine release syndrome, ischemia due to hypercoagulability, and thrombotic microangiopathy due to SARS-CoV-2 infection [19,20,21,22,23]. However, all the aforementioned findings are just hypotheses of a possible relationship between COVID-19 and AAC, since there are no large studies with robust data supporting a definite causal relationship between these two pathologies.

## 4. Types of AAC

Pathologically, four types of AAC are described [1]:

1. Simple cholecystitis (inflammation of the gallbladder with no other complications).
2. Acute suppurative cholecystitis (cholecystitis complicated by the presence of pus in the gallbladder lumen).
3. Gangrene cholecystitis (cholecystitis complicated by gallbladder wall necrosis).
4. Gallbladder perforation (cholecystitis complicated by gallbladder wall rupture).

Aetiologically, the inflammation occurring in ACC can be due to the following causes [1]:

1. Mechanical causes (due to increased pressure in the gallbladder lumen resulting in the compression and ischemia of its wall and mucosa).
2. Chemical causes (phospholipases act on the lecithin of bile, producing hemolytic lecithin, resulting in chemical inflammation).
3. Bacterial causes (presence of microorganisms in the gallbladder).

## 5. AAC Pathogenesis

AAC development involves an interplay between several pathological processes. When AAC is associated with infectious agents, its pathogenesis can be divided into two categories: (1) AAC in critically ill patients and (2) AAC in patients with a non-critical illness [2].

### 5.1. AAC in Critically Ill Patients

#### 5.1.1. Bile Stasis

Bile stasis plays a crucial role in the pathogenesis of AAC in critically ill patients. This can be induced by TPN and prolonged fasting, volume depletion, opioid analgesics, hormones, and certain drugs that impair either the viscosity or the mobility of the gallbladder smooth muscle, causing cholestasis [24,25,26,27,28,29,30]. Additionally, a modified lipid metabolism in type II diabetes and cerebrovascular disease and mechanical ventilation with positive end-expiratory pressure (PEEP) > 7 cm H_2_O also lead to cholestasis [31,32]. Bile stasis causes direct injury to the gallbladder mucosa due to changes in the bile’s chemical composition (e.g., lysophosphatidylcholine) [33].

#### 5.1.2. Gallbladder Ischemia–Reperfusion Injury

The gallbladder is mainly supplied by the cystic artery, a terminal artery, so it is vulnerable to ischemia. Tissue hypoxia may be caused by septic shock, hypovolemic shock, trauma, burns, and cardiovascular surgeries. Objective evidence of the role of ischemia in AAC has been provided by microangiography showing a poor and irregular capillary network in AAC, in contrast with acute calculous cholecystitis (ACC), where a dense vessel network and dilated arterioles are depicted [31,34,35,36,37,38].

### 5.2. AAC in Non-Critically Ill Patients

#### 5.2.1. Direct Invasion

Several microorganisms can infiltrate the gallbladder’s epithelium and initiate an inflammatory process. They are usually identified by a combination of the histopathology of the gallbladder wall, molecular techniques, electron micrographs, and bile cultures. The overuse of opioids during GI surgeries, as well as the sphincter of Oddi being subjected injury and spasming, can lead to infection. Moreover, invasive procedures like endoscopic retrograde cholangiopancreatography (ERCP) have a risk of bacterial infection. Sepsis and systemic infection can also induce AAC by gallbladder bacterial colonization through blood circulation. Additionally, infections of the surrounding organs can transmit bacteria directly into the gallbladder [24,39,40,41,42,43,44,45,46,47,48,49,50,51,52,53,54,55,56,57,58,59,60,61,62].

#### 5.2.2. Vasculitis

Vasculitis is another mechanism that causes gallbladder injury by inducing local ischemia and, subsequently, cell death and gallbladder necrosis. Several viruses have been reported to affect the gallbladder through immune-mediated necrotizing vasculitis. Antibody dependent cell-mediated cytotoxicity or complement-dependent cytotoxicity are considered possible mechanisms, while others [63,64,65,66] have stated that AAC can be caused by the destruction of the endothelium, causing the extravasation of fluid and leading to gallbladder wall edema or nonocclusive thrombosis [67,68].

#### 5.2.3. Obstruction

The biliary tract may be obstructed by intrinsic or extrinsic factors, leading to different degrees of obstruction and, subsequently, AAC. Increased intraluminal pressure and increased gallbladder wall tension lead to ischemia and AAC [69,70,71]. Extrinsic compression and the obstruction of the biliary tree may arise from the formation of hydatid cysts during the course of echinococciasis or from portal lymphadenitis during the course of infectious diseases like Epstein–Barr virus (EBV) disease [72,73].

#### 5.2.4. Anatomical Abnormalities of the Biliary Tract System

Congenital abnormalities of the biliary system, for example, biliary cysts, torsion of the gallbladder, enlarged gallbladder neck, duct diameter narrowing, or a completely intrahepatic gallbladder, may impair the ability to empty the bile ducts. This leads to cholestasis, followed by an increased risk of AAC [57,74,75,76,77].

#### 5.2.5. Other Risk Factors

Other risk factors that increase the risk of AAC are immunodeficiencies, atherosclerosis, vasculitis, diabetes mellitus, stroke, arterial hypertension, factors reducing vagal tone, bone marrow transplantation, interleukin-2 therapy, and lymphokine-activated killer cell therapy [35,78,79,80,81,82,83].

#### 5.2.6. Sequestration

Sequestration explains the pathogenesis of ACC in malaria infections and especially during Plasmodium falciparum infection. Infected erythrocytes lose their normal shape due to the appearance of protrusions (knobs) on their surface. This deformation causes erythrocyte cells to adhere to each other and to the vessel walls, resulting in microcirculatory obstruction and ischemia [84,85,86,87].

#### 5.2.7. Epstein–Barr Virus (EBV)

EBV has been reported as the most frequent infectious cause of AAC. The exact pathophysiological mechanism remains unclear, with the direct invasion of the gallbladder’s epithelial cells, vasculitis, and the extrinsic blockage of the cystic duct by inflamed and enlarged lymph nodes all having been proposed. It is a fact, however, that even after cholecystectomy and the in situ hybridization of the removed tissue, the virus may not be revealed [40,73,87,88,89].

## 6. AAC Pathogenesis in COVID-19

The pathogenesis of AAC during COVID-19 infection still remains vastly unknown. Critical illness, mechanical ventilation, and prolonged total parenteral nutrition (TPN) are considered the usual triggers. SARS-CoV-2 enters cells through the angiotensin-converting enzyme 2 (ACE2) receptors. Because ACE2 receptors are highly expressed in the biliary tree and the vascular endothelium of the gallbladder, the direct invasion of these structures is considered a possible pathophysiological mechanism. Alternatively, SARS-CoV-2 can infect small intestinal enterocytes which express ACE 2 receptors and enter the gallbladder through the bile recirculation process. It can also be supposed that the virus may spread to extrapulmonary organs including the gallbladder and give typical clinical manifestations [21,90,91,92]. It should be noted, however, that in most cases, the virus has not been detected locally in the bile or tissue samples of affected patients, except for in two cases [19,93].

A few theories trying to explain possible connections between SARS-CoV-2 and AAC have been defined. One such theory considers that the alterations to the immune system due to the SARS-CoV-2, the systemic inflammation that follows, and the subsequent treatment applied activates proinflammatory pathways that cause late-onset cholecystitis [94]. Moreover, the coagulopathy and prothrombotic state caused by the coronavirus can cause small-vessel thrombosis and gallbladder wall ischemia [95,96,97,98]. Furthermore, SARS-CoV-2 can directly affect the gallbladder’s epithelial cells because of the high expression of ACE2 receptors among them [99]. In a study by Bozada-Gutiérrez et al., a histopathological examination showed ischemia and necrosis (because of thrombosis in the small vessel), hemorrhagic changes, and acute inflammatory infiltrates in the gallbladder wall, accompanied by acute peritonitis [100].

## 7. Worldwide Cases of AAC in COVID-19

Several reports in the literature have described the presentation of acute cholecystitis in COVID-19 patients, with most of them being case reports and letters to the editor and some case series (Table 1) [10,19,20,21,22,91,92,94,95,100,101,102,103,104,105,106,107,108,109,110,111,112,113,114].

Bruni et al., described the first case of histopathological findings of an acute ischemic gangrenous cholecystitis as a late complication in a COVID-19 [95].

Several other cases pointed out the presence of gangrenous AAC [21,91,101]. In a cohort study by Bozada et al., all patients had cholelithiasis except for one with AAC [100]. Different pathophysiological mechanisms, all of which would need further analysis, could be hypothesized here. However, all patients required cholecystectomy, irrespective of the aetiology of the cholecystitis. In the ChoCO-W prospective observational global study, there was no difference in AAC between non-COVID and COVID patients (3.9% vs. 4.6%, *p* = 0.18); cholecystitis with necrosis/gangrene was more common in the COVID group (40.7% vs. 22.3%), and mortality was higher in the COVID group (13.4% vs. 1.7%) [110].

Cirillo et al. [102] presented a case of acalculous hemorrhagic cholecystitis in a COVID-19 patient that required an emergency cholecystectomy. However, this is a very rare finding, and establishing an association with COVID-19 would require larger studies.

Regarding acute cholecystitis management, antibiotics [117], percutaneous cholecystostomy [118,119], and laparoscopic or open cholecystectomy [117,120] have been applied. When medical treatment with antibiotics only or cholecystostomy are considered insufficient, urgent cholecystectomy is indicated [101,121,122] (Table 1).

## 8. Diagnosis of ACC in COVID-19

### 8.1. Signs and Symptoms

AAC usually presents with a fever of unknown origin and persistent or intermittent right upper abdominal pain, together with other nonspecific signs of GI disease, like nausea, vomiting, and jaundice. When complicated by gallbladder perforation, right epigastric tenderness, a positive Murphy’s sign, a palpable right epigastric mass, and peritoneal irritation usually arise. All these symptoms and signs cannot be evaluated in critically ill intubated patients, and this is one of the reasons for delayed diagnoses in such circumstances [123].

### 8.2. Laboratory Investigations

Several laboratory abnormalities occur in AAC; however, they are nonspecific for the disease and should be carefully taken into account and evaluated in the context of the other clinical and imaging findings. Such abnormalities include higher than normal levels of white blood cells, neutrophils, and C-reactive protein; an increase in aminotransferase; mild-to-moderate bilirubin elevation, small blood amylase elevation, and positive blood cultures [123].

### 8.3. Imaging

Imaging plays a crucial role in AAC diagnosis, taking into account the predisposing factors, clinical situation, and laboratory findings.

B-scan ultrasound examination of the upper abdomen is usually the first-line modality, since it is simple, inexpensive, easy to perform (even at bedside), widely available, and effective (specificity 30–100%, sensitivity 30–100%), with no obvious contraindications. The diagnostic criteria for AAC are as follows: (1) positive Murphy’s sign upon ultrasound, (2) no gallbladder stones, (3) gallbladder dilatation (longitudinal diameter ≥ 8 cm and transverse diameter ≥ 5 cm), (4) gallbladder wall thickening (gallbladder wall ≥ 3.5 mm, depicted as “bilateral” or “double-walled” sign), (5) peritoneal effusion and biliary-type gallbladder drainage, (6) echoes occurring in the gallbladder cavity or wall, and (7) bile (bile sludge may be present) [124].

CT scan of the abdomen is the second most frequently used modality, typically deployed when an ultrasound cannot reach a definite conclusion or to confirm the findings of the ultrasound. When both are used, sensitivity and specificity significantly increases. The diagnostic criteria for the CT scan are as follows: (1) no stones in the gallbladder neck or cystic duct and thickened (≥0.3 cm) and enhancing wall; (2) pericholecystic fat stranding with or without pericholecystic fluid and reactive enhancement in the adjacent liver; (3) in cases of gangrenous cholecystitis, nonenhancing wall, sloughed membranes, mural striation, irregular enhancing wall with defect and pericholecystic fat stranding and fluid are noted; (4) when the gallbladder is perforated, focal defect in the gallbladder wall accompanied by pericholecystic fluid or hepatic abscesses [125,126,127].

Magnetic resonance cholangiopancreatography (MRCP) is another non-invasive imaging option. This is a simple and safe exam that depicts the bile pancreatic duct system. It can also provide 3D and multiangle images of the biliary tract system to detect anatomical abnormalities. However, it is more expensive, complicated, and not widely available, so its use in clinical practice remains limited [128].

Endoscopic retrograde cholangiopancreatography (ERCP) is an invasive method that provides direct imaging of the biliary tree and also the possibility to act therapeutically. However, it is more associated with pancreatitis and has limitations in anatomical abnormalities of the biliary tract, so it also not frequently used [1].

Another, more sophisticated, imaging technique is hepatobiliary dynamic imaging (HIDA). In HIDA, hepatic bilirubin distribution is stimulated by the intravenous injection of acetanilide iminodiacetic acid compounds radiolabeled by imaging agent 99 m TC-EHIDA. The imaging agent is absorbed by liver cells, secreted into bile, and then excreted into the intestinal tract, and it can be observed through a flashing dynamic imaging camera to understand the morphological and functional structure of the liver and gallbladder. The test can take hours to reach a final conclusion; it is not useful for seriously ill patients and is not widely available, so its use is very limited [129].

### 8.4. Pathology

AAC, unlike calculous cholecystitis, is usually complicated by necrosis of the gallbladder tissue because of blood circulation disorder and blood stasis. Histopathology reveals a degenerated and necrotic mucosal epithelium of the gallbladder, the formation of multiple erosive ulcers, and infiltration by many neutrophils and other inflammatory cells [130].

## 9. Differential Diagnosis of AAC in COVID-19

Diagnosing AAC is difficult, and clinical findings may be misleading sometimes, especially in critically ill patients and in patients with many other comorbidities. The main conditions that are involved in the differential diagnosis of AAC and may mimic its clinical manifestations are other gastrointestinal pathologies like cholangitis, pancreatitis, hepatitis, sepsis with biliary tract infection, acquired immunodeficiency syndrome (AIDS), cholangiopathy, total parenteral nutrition (TPN), alcohol-associated liver disease, ischemic bowel disease, bowel perforation, renal pathologies like nephrolithiasis, and cardiovascular disease (CVD) [131,132,133].

Clinically, in COVID-19 patients, making a clear distinction between acute acalculous cholecystitis and acute calculous cholecystitis is very difficult because the symptoms are similar and only imaging (ultrasound or CT) can help differentiate between the two conditions. As reported by the ChoCo-W, even in COVID-19 patients, gallstone cholecystitis was more frequent, with no difference in symptoms between AAC and ACC.

Especially for CVD, AAC may present like angina, myocarditis/pericarditis, and myocardial infarction with elevated troponin levels and ECG changes (T wave inversion, ST segment elevation, or depression), probably due to coronary vasospasms induced by a vagally mediated reflex or by decreased coronary blood flow due to distention of the common bile duct [131,132,133].

Imaging methods that can be combined with abdominal ultrasound and abdominal CT scan are usually the modalities that will differentiate between AAC and most of the other gastrointestinal or renal pathologies. As CVD is measured via serial troponin measurements (to record its kinetics), serial ECGs (to monitor T wave and ST segments changes), echocardiography (to exclude segmental myocardial wall motion abnormalities and pericardial effusion), and coronary angiography in very high-risk patients for CVD will be needed when AAC diagnosis remains vague after the aforementioned exams in order to identify early cardiovascular pathology [131,132,133].

For critically ill patients, the use of diagnostic laparoscopy and laparotomy when AAC cannot be excluded by non-invasive methods is also recommended.

## 10. Treatment of AAC in COVID-19

The severity of AAC and subsequent treatment are largely based on the 2018 Tokyo Guidelines, which take into account predisposing factors; the Charlson Comorbidity Index score; and the American Society of Anesthesiologists-physical status score [134]. There are three levels of severity, ranging from I to III, with III being the most serious. Grade III (severe) AAC is associated with organ or systemic dysfunction, including cardiovascular dysfunction, neurological dysfunction, respiratory dysfunction (PaO2/FiO2 ratio < 300), kidney dysfunction (oliguria or creatinine > 2.0 mg/dL), hepatic dysfunction (PT-INR > 1.5), and hematological dysfunction (platelet count < 100,000/mm^3^). Grade II (moderate) AAC involves an elevated WBC count (>18,000/mm^3^), palpable tender mass in the right upper abdominal quadrant, duration of symptoms > 72 h, and marked local inflammation (gangrenous cholecystitis, pericholecystic abscess, hepatic abscess, biliary peritonitis, and emphysematous cholecystitis). Grade I (mild) AAC is defined as acute cholecystitis in an otherwise healthy patient with no organ dysfunction and mild inflammatory changes in the gallbladder [121].

Mild AAC in the early stage is managed conservatively. After nonsurgical treatment, the recurrence rate is lower in AAC than in ACC (2.7 vs. 23.2%). Severe AAC requires surgical treatment. The incidence of gangrenous cholecystitis is higher in AAC [135,136,137]. In contrast early surgical treatment is recommended for patients with ACC.

In AAC, the mortality rate after surgery in non-COVID-19 patients is 9–75%, higher than that for calculous cholecystitis. However, this also depends on the severity of the underlying disease and is usually higher when there are systematic organ lesions rather than just hepatobiliary complications [138,139].

### 10.1. Conservative Treatment

Conservative treatment is initially applied in mild stages of AAC in early-diagnosed patients who are in a good general condition. Conservative measures include fasting, gastrointestinal decompression, antibiotics, electrolyte and acid-base homeostasis, symptomatic support treatment, and primary disease management [136]. In order to select the most appropriate antibiotic regimen, it is crucial to carefully take into account all laboratory markers, vital signs, and overall condition. Biliary tract infections are usually caused by Gram-negative bacteria and third-generation cephalosporins, and aminoglycosides are more often used. If conservative treatment is deemed unsuccessful in controlling the inflammation, surgical treatment is the next step to diminish the risk of gallbladder necrosis and perforation.

### 10.2. Cholecystectomy

Cholecystectomy, whether laparoscopic, open, or partial cholecystectomy, is the preferred treatment for AAC, minimizing trauma. According to the 2018 Tokyo Guidelines, the surgical technique should be individualized based on the surgical risk. Laparoscopy offers safety benefits like less painful incision sites, shorter hospital stays, shorter recovery periods, and improved quality of life [134].

### 10.3. Drainage

For critically ill patients unable to tolerate anesthesia and surgery, ultrasound-guided percutaneous cholecystostomy (PC) is a good alternative. During this procedure, called percutaneous transhepatic gallbladder drainage (PTGD), a PC catheter is placed in the gallbladder through the liver and the abdomen. PTGD is generally the first choice in clinical practice [140,141,142]. The advantages of PTGD are as follows: (1) it relieves the pressure in the gallbladder immediately, resulting in a decline in inflammation; (2) it is less traumatic and has fewer anesthetic complications compared to surgery; (3) the operation time is short, meaning that the recovery time is also short, and there are few postoperative complications; and (4) it can be used as the only treatment.

Especially for elderly patients, PTGD is also a safe and effective treatment method with proven efficacy and few complications [140]. After placing the PC tube, the color, drainage, and bile characteristics should be checked regularly to avoid the prolapse and contortion of the PC tube and to evaluate liver function, focusing on the presence of cholestasis. However, there is still a debate regarding the optimal timing of PC tube removal (at 4 or 7 days) [135,143].

A new and effective alternative gallbladder drainage procedure in AAC patients is also endoscopic transpapillary gallbladder drainage under ERCP (involving endoscopic naso-gallbladder drainage and gallbladder stenting [EGBS] and endoscopic ultrasound-guided gallbladder drainage [EUS-GBD]) [144]. During this procedure, a lumen-apposing metal stent (LAMS) is placed between the duodenal bulb or the gastric antrum and the gallbladder during an endoscopic ultrasound. The two sides of the stent are fixed to the gastrointestinal tract and the gallbladder, respectively, to establish a safe passage for internal drainage. This method can be applied in cases of gallbladder enlargement that are difficult to manage with direct surgical resection and in cases with an increased risk for duodenal or gastric perforation. It can also be applied as a form of palliative care for patients at an extreme or very high risk of surgery or those who have a short life expectancy [143,144,145,146,147].

## 11. Outcomes of AAC in COVID-19

As seen in Table 1, most cases of AAC in COVID patients were managed successfully and recovered. Death usually occurred in high-risk or critically ill patients or when complications occurred. However, in the ChoCO-W prospective observational global study (acute cholecystitis comparison between 180 COVID and 2412 non-COVID patients), mortality was higher in the COVID group (13.4% vs. 1.7%) [110]. When distinguishing between non-ICU-associated COVID-19 AAC (non-ICU–AAC) and ICU-associated COVID-19 AAC (ICU-AAC), we can observe that most cases were non-ICU AAC (n = 29) rather than ICU-AAC (n = 19), with four deaths versus seven deaths, respectively (excluding the ChoCo-W registry that included acute cholecystitis in general). Based on these reports, if we could just try to provide an impression (no definite evidence at all) about mortality, it looks like the mortality rate is lower in non-ICU-AAC patients (13.8%) versus ICU-AAC patients (36.8%). ICU-AAC onset was usually after a long stay in the ICU including mechanical ventilation, increasing the vulnerability of these patients and leading to an increased mortality rate.

## 12. Conclusions

AAC could be considered a possible complication of COVID-19 disease, especially in patients with concomitant comorbidities or other risk factors for AAC. The pathogenesis of AAC in COVID-19 has not been fully elucidated, and more evidence is needed to understand the effect of SARS-CoV-2 on the hepato-biliary system. Initial conservative management followed by percutaneous cholecystostomy when it fails looks to be the most feasible option during AAC cases with COVID-19. Cholecystectomies, laparoscopic or open, are reserved in case of complications (perforation, gangrene). Although the outcomes are usually favorable, especially in non-critically ill patients, morbidity and mortality are increased in critically ill patients and when AAC is complicated by gangrene and/or perforation.

## Figures and Tables

**Table 1:** Acute (acalculous) cholecystitis in COVID-19 patients.

Author	Patient Gender—Comorbidities	Age	Time of Onset—Clinical Characteristics	Symptoms—Findings—Diagnosis—Severity	Management	Outcome
**Non-ICU-associated COVID-19 AAC**						
Balaphas, 2020 [19]	Female	84	AAC onset: 4 days after fever Sepsis due to pyelonephritis—ARDS due to COVID-19—10 days from hospital admission to death	Right upper quadrant pain, positive Murphy, increased CRP, ARDS. Ultrasonography and CT scan: no gallbladder perforation. Histological analysis of the gallbladder did not demonstrate any inflammation, but quantitative reverse transcriptase PCR (qRT-PCR) revealed the presence of SARS-CoV-2 in all 3 sampled regions of the gallbladder wall.	Supportive care, ceftriaxone, metronidazole, laparoscopic cholecystectomy	Death (multiorgan failure)
Balaphas, 2020 [19]	Male CKD-dialysis, DM2, ArtHTN, modAoS	83	AAC onset: 5 days after fever Respiratory symptoms due to COVID-19	Right upper quadrant pain, positive Murphy sign, increased CRP, white blood cells, hepatic enzymes. Ultrasonography: 4 mm thickening of the gallbladder wall, presence of peri-vesicular liquid, absence of gallstones.	Conservative management with ceftriaxone and metronidazole	Recovered
Ying, 2020 [22]	Female	68	AAC onset: after 10 days of hospitalization for COVID-19 pneumonia 6 days of fever—COVID-19 pneumonia	Right upper quadrant pain, diarrhea, Murphy’s sign after 10 days of hospitalization, fever, elevated CRP; bile was negative for SARS-CoV-2. CT scan: distended gallbladder, hyperplasia of the gallbladder wall, and biliary sludge; CT scan did not show gallstones in the gallbladder.	Ultrasound-guided percutaneous transhepatic gallbladder drainage (PTGD), antibacterial and anti-viral lopinavir/ritonavir combined with human interferon alfa-1b inhalation	Recovered
Lovece, 2020 [94]	Male	42	AAC onset: after 10 days of hospitalization for COVID-19 pneumonia 7 days of fever—hypoxemia—CPAP	Nausea and upper-quadrant abdominal pain, afebrile, diffuse abdominal tenderness, rebound pain. Ultrasound and CT scan: absence of contrast enhancement of the gallbladder and microperforation of the fundus	Emergency laparoscopic cholecystectomy	Recovered
Bozada-Gutiérrez, 2022 [100]	Females: 4 Males: 6	Mean age: 47.1 (range 20–74)	AAC onset: not defined COVID-19 pneumonia (n = 6) Asymptomatic (n = 4) ICU admission after AAC (n = 5) Parkland grading scale 3 (n = 2) 4 (n = 2) 5 (n = 6)	Right upper quadrant pain (n = 10), right upper quadrant mass (n = 6), and positive Murphy’s sign (n = 10). All patients underwent chest computed tomography (CT) scans prior to surgery. Also, all patients underwent gallbladder ultrasound. Only one acalculous.	Two patients required preoperative endoscopic retrograde cholangiopancreatography (ERCP). All patients were treated with urgent/early Lap-C. Eight surgeries were completed via laparoscopy, and two patients required conversion to open cholecystectomy due to operative difficulty.	Death: 1 Recovered: 9
Asti, 2020 [101]	F: 86 M: 72 M: 40	NR	AAC onset: not defined; all recovering from COVID-19 pneumonia	Acute abdomen CT: acute acalculous cholecystitis.	Emergency laparoscopy confirmed gallbladder gangrene in all associated with fundic microperforation in the youngest patient, and cholecystectomy was completed without complications and no conversion	Recovered
Cirillo, 2020 [102]	Male	79	AAC onset: after 7 days of hospitalization for COVID-19 pneumonia COVID-19 during hospitalization in a rehabilitation clinic after hip replacement for fracture	Anemia/bleeding, abdominal tenderness on the right upper quadrant. CT scan: perforated acalculous gallbladder.	Emergency cholecystectomy	Recovered
Barbachowska, 2022 [104]	Male living-donor kidney transplantation (mother) in 2017 due to end-stage kidney disease in course of IgA nephropathy	34	AAC onset: recurrent fever up to 39.5 °C lasting for 3 weeks—COVID-19 pneumonia	Chronic fatigue, back pain, bloating, nausea, pain in upper quadrant of the abdomen, loss of appetite. CT scan, cholangio-MRI: acalculous cholecystitis with presence of pericholecystitis and increased density and edema of surrounding tissues.	LMWH, antibiotics	Recovered
D’ Introno, 2022 [92]	Male DM2, artHTN	50	AAC onset: after 6 days of generalized abdominal pain, low fever, nausea, and vomiting Dehydration, COVID-19 positive with no respirator symptoms	Tenderness on the right upper quadrant and in the epigastrium, positive Murphy’s sign. Ultrasonography and CT scan: gangrenous gallbladder with perihepatic fluid.	Initial antibiotics (piperacillin/tazobactam) failed, laparoscopic cholecystectomy	Recovered
Deif, 2022 [105]	Male DM	55	AAC onset: not defined COVID-19 pneumonia—ambulatory	Abdominal pain. Ultrasound: acute cholecystitis.	Percutaneous cholecystostomy	Recovered
Liapis, 2022 [107]	Male	53	AAC onset: after COVID-19 infection for 10 days Fever spikes, mild dyspnea, non-productive cough	Epigastric pain and fever. Right upper quadrant tenderness and a positive Murphy’s sign. Ultrasound and CT scan: gangrenous gallbladder with no sign of perforation.	Laparoscopic cholecystectomy and antibiotics	Recovered
Hajebi, 2022 [108]	Female artHTN, appendectomy	86	AAC onset: 1 day after generalized abdominal pain COVID-19 pneumonia—ambulatory	Generalized abdominal pain, vomiting, loss of appetite, weight loss, severe tenderness in right upper and lower quadrants, epigastrium, and hypogastrium. Ultrasound and CT scan: gallbladder distension with surrounding fluid.	Open cholecystectomy	Recovered
Hajebi, 2022 [108]	Male	82	AAC onset: not defined COVID-19 pneumonia—ambulatory	Abdominal pain and tenderness in right upper quadrant, vomiting. CT scan: emphysematous cholecystitis.	Open cholecystectomy	Recovered
Futagami, 2022 [115]	Male CKD on dialysis	42	AAC onset: after 7 days in ward for fever and cough COVID-19 pneumonia—hypoxia—increased oxygen administration by non-rebreather mask	Abdominal pain. CT scan: moderate grade AAC.	Percutaneous transhepatic gallbladder drainage (PTGBD), antibitiotics and laparoscopic cholecystectomy when patient was COVID (−)	Recovered
Alam, 2021 [109]	Female	84	AAC onset: after 2 days of generalized abdominal pain, vomiting, and diarrhea COVID-19 pneumonia—intubation due to septic shock	Generalized abdominal pain, positive Murphy’s sign, vomiting, diarrhea, altered general status, respiratory distress, desaturation, hypotensive. CT scan: Acute ischemic gangrenous cholecystitis.	Conservative initially and scheduled for percutaneous drainage. Cardiac arrest.	Death (septic shock)
De Simone, 2022 ChoCo-W study [110]	The aim of the ChoCO-W global prospective study was to compare the clinical course, biological and radiological findings, and clinical outcomes of AC in patients who have COVID-19 disease with those who do not have it	COVID: 180. Non-COVID: 2412. Age: 63.93 (15.8) vs. 96 (53.3%).	AAC onset: not defined PCR-positive COVID-19 patients with acute cholecystitis irrespective of clinical status	Acalculous cholecystitis: COVID 8 (4.6%) vs. non-COVID 93 (3.9%) *p* = 0.18.	All interventions	Mortality rate was 13.4% (24/180) in the COVID group and 1.7% (40/2412) in the non-COVID group (*p* < 0.0001).
Abaleka, 2021 [111]	Female Afib, HF, pacemaker, asthma	76	AAC onset: dry cough and dyspnea simultaneously Acute hypoxic respiratory failure due to COVID-19 pneumonia—oxygen via nasal cannula	Right upper quadrant abdominal pain, nausea, vomiting, positive Murphy’s sign. Ultrasound: increased gallbladder wall thickness with mild pericholecystic fluid collection.	Conservatively—remdesivir, vitamin supplements, piperacillin/tazobactam, dexamethasone, apixaban. Scheduled for elective cholecystectomy.	Recovered
Hassani, 2020 [112]	Male artHTN, IHD-CABG	65	AAC onset: abrupt with abdominal pain COVID-19 pneumonia—ambulatory	Abdominal pain, intermittent shaking chills without fever, positive Murphy’s sign, vomiting. Ultrasound: increased gallbladder wall thickness.	Conservatively—favipiravir, intravenous crystalloid resuscitation, analgesics.	Recovered
Alhassan, 2020 [91]	Female	40	AAC onset: after 2 days of high-grade fever, generalized body aches, nausea, and moderate right hypochondrium pain COVID-19 pneumonia—ambulatory	Fever, generalized body aches, nausea, moderate right hypochondrium pain, positive Murphy’s sign. Ultrasound: thickened gall bladder wall, surrounding pericholecystic fluid, and minimal free fluid in the abdomen and the pelvis.	Conservatively. Antibiotics.	Recovered
Safari, 2020 [113]	Female chronic kidney disease, valvular heart disease, hypertension	75	AAC onset: not defined COVID-19 positive—only cough—ambulatory	CT scan: acute cholecystitis—gallbladder empyema.	Unsuccessful nonoperative management for 48 h; she was scheduled for laparoscopic cholecystectomy	Death
Basukala, 2021 [114]	Male DM	47	AAC onset: after 6 days in COVID-19 ward COVID-19 positive—fever, cough, dyspnea—ambulatory	Right upper abdominal pain; positive Murphy’s sign was present. Initial ultrasound: acute cholecystitis.	Initial conservative management failed. Patient developed gallbladder perforation. Emergency exploratory laparotomy and cholecystectomy. Histopathological report revealed gallbladder perforation at the fundus upon gross examination and ischemic necrosis of gallbladder mucosa upon microscopic examination.	Recovered
**ICU-associated COVID-19 AAC**						
Puig, 2021 [20]	Male ex-smoker, ArtHTN	65	AAC: 7 days after extubation 10 days fever—ARDS due to COVID-19—NIMV—intubation—extubation after 5 days—HFNC—reintubation due to septic shock—extubation and HFNC—re-intubation due to lower gastrointestinal bleeding—tracheotomy—weaning	Tachypnea together with intense abdominal pain with guarding in the right upper quadrant, septic shock, massive bilateral pulmonary embolism, ischemic colitis. CT scan.	Ultrasound-guided percutaneous cholecystostomy, ertapenem, enoxaparin	Recovered
Franch-Llasat, 2022 [103]	Male	73	AAC onset: after 2 days in ward, following 41 days in the ICU ARDS due to COVID-19—intubation—mechanical ventilation for 34 days—enteral nutrition for 41 days—extubated	Two days after ICU discharge: abdominal pain, fever, elevated CRP. Ultrasound: hydropic gallbladder with thickened walls and incipient necrosis.	Due to the patient’s frail condition, percutaneous drainage was performed, and antibiotics were administered.	Recovered
Franch-Llasat, 2022 [103]	Male	42	AAC onset: after 2 days in a rehabilitation center, following 36 days in the ICU ARDS due to COVID-19—intubation—mechanical ventilation for 35 days—enteral nutrition for 36 days—extubated	Twelve days after extubation: persistent abdominal pain. CT scan: distended gallbladder with poorly defined walls suggestive of edema.	Laparoscopic cholecystectomy, antibiotics. Staphylococcus warneri was isolated in the bile.	Recovered
Franch-Llasat, 2022 [103]	Male	67	AAC onset: after 20 days in a rehabilitation center, following 70 days in the ICU ARDS due to COVID-19—intubation—mechanical ventilation for 63 days—enteral nutrition for 70 days—extubated	Twenty days after extubation: abdominal pain, vomiting, leukocytosis. Ultrasound: distended gallbladder with an edematous wall.	Laparoscopic cholecystectomy, antibiotics, emergency reoperation for hemorrhagic shock secondary to bleeding from the cystic artery	Recovered
Deif, 2022 [105]	Male IHD. AKI	72	AAC onset: after mechanical ventilation for 40 days COVID-19 pneumonia	Fever. Ultrasound: acute cholecystitis, distended thick-walled gall bladder with biliary dilatation.	Percutaneous cholecystostomy	Death
Deif, 2022 [105]	Male Jaundice. AKI (on dialysis)	61	AAC onset: after mechanical ventilation for 2 days COVID-19 pneumonia	Fever. Ultrasound: cholangitis and cholecystitis.	Percutaneous cholecystostomy	Death
Wahid, 2020 [106]	Female artHTN, DM2, hypothyroidism	60	AAC onset: after 44 days of hospitalization including mechanical ventilation ARDS due to COVID-19 pneumonia	Fever, positive Murphy’s sign. Ultrasound and CT scan: gallbladder distension, biliary sludge.	Cholecystostomy tube and antibiotics	Recovered
Wahid, 2020 [106]	Male	68	AAC onset: after 67 days of hospitalization including mechanical ventilation ARDS due to COVID-19 pneumonia	Positive Murphy’s sign. Ultrasound and CT scan: gallbladder distension, biliary sludge.	Cholecystostomy tube and antibiotics	Recovered
Chen, 2022 [116]	F: 3 (33%) M: 6 (67%) 9 cases vs. 203 controls	60 (52, 68)	AAC onset: not defined Mechanical ventilation: 8 (89%)	Ultrasound severity: not defined.	Percutaneous cholecystostomy	Death: 5 (56%) Recovered: 4 (44%)
Mattone, 2020 [21]	Male ex-smoker	66	AAC onset: 49 days after intubation in ICU ARDS due to COVID-19 pneumonia—intubated	At 49th day of hospitalization: right upper quadrant abdominal pain, nausea, vomiting, tender abdomen, positive Murphy’s sign. CT scan: gallbladder wall thickening, no gallstones.	A percutaneous transhepatic biliary drainage (PTBD) under ultrasound control of gallbladder. A sample of bile was tested for SARS-CoV-2 RNA, and it was negative. Laparoscopic cholecystectomy after 3 days with no improvement.	Recovered
Bruni, 2020 [95]	Male	59	AAC onset: after 32 days in ICU ARDS due to COVID-19—intubated—mechanical ventilation for 8 days	At 32nd day of hospitalization: abdominal pain without signs of peritonism, increased inflammatory and cholestasis indexes. CT scan: gallbladder perforation.	Laparotomic cholecystectomy	Recovered
